# A longitudinal cohort study of adolescent elite footballers and controls investigating the development of cam morphology

**DOI:** 10.1038/s41598-021-97957-2

**Published:** 2021-09-17

**Authors:** Scott Fernquest, Antony Palmer, Mo Gimpel, Richard Birchall, John Broomfield, Thamindu Wedatilake, Hendrik Dijkstra, Joanna Burchall, Thomas Lloyd, Claudio Pereira, Simon Newman, Andrew Carr, Sion Glyn-Jones

**Affiliations:** 1 Botnar Research Centre, Old Road, Oxford, OX3 7LD UK; 2 Nuffield Department of Orthopaedics, Rheumatology, and Musculoskeletal Sciences, University of Oxford, Oxford, UK; 3 Southampton Football Club, Southampton, UK; 4 Aspetar, Qatar Orthopaedic and Sports Medicine Hospital, Doha, Qatar; 5 Weill Cornell Medicine Qatar, Doha, Qatar; 6 Department of Continuing Education, University of Oxford, Oxford, UK

**Keywords:** Bone, Cartilage, Risk factors, Lifestyle modification

## Abstract

Cam morphology describes an asphericity of the femoral head that develops during adolescence, is highly prevalent in athletes, and predisposes individuals to future osteoarthritis. However, it’s aetiology remains poorly understood. The aim of this study was to perform 3-year longitudinal follow-up of a control population and football club academy cohort to compare the change in hip and growth plate anatomy between athletes and controls. MRI and questionnaires were used to characterise change in hip and growth plate anatomy and quantify activity levels. 121 male academy footballers and 107 male and female controls participated at baseline. Footballers experienced significantly greater increases in femoral head asphericity (4.83 degrees (95% CI: 2.84 to 6.82), p < 0.001) than controls. A positive correlation existed between activity levels and change in femoral head morphology (coefficient 0.79, p  ≤  0.001). Greatest morphological change occurred in individuals aged 11–12 years at baseline, with no significant change in individuals aged 14 years and older at baseline. Cam morphology development was secondary to soft tissue hypertrophy and lateral growth plate extension. In conclusion, excessive loading of the hip joint through exercise prior to 14 years of age may result in growth plate adaptations causing cam morphology. Potential interventions may include training type and load modification in young adolescent football players.

## Introduction

Cam morphology describes an asphericity of the femoral head that develops during adolescence^1,2^. It is present in approximately a quarter of the general population and over half of elite athletes^3^, being more common in males than females^4^. Cam morphology is a strong risk factor for the development of hip pain, osteoarthritis, and future total hip arthroplasty^5^. An improved understanding of cam morphology development is required to determine whether cam formation is preventable or modifiable.

Preventing the development of cam morphology formation requires the identification of individuals at risk, which studies to date suggest are males participating in sports such as football at a high or elite level during adolescence^6,7^. However, it is not known how to define high level sport or activity in general, and at what age or stage of adolescence hips are most susceptible to developing cam morphology^8–10^. These uncertainties limit the development of strategies to prevent cam formation. Further uncertainties revolve around the mechanism of cam morphology formation. Subclinical Slipped Upper Femoral Epiphysis (SUFE) as the dominant aetiological factor for cam morphology has been brought into question by recent studies^11^. Epiphyseal extension along the anterosuperior femoral neck is an increasingly recognized mechanistic phenomenon in athletes with cam morphology^12^. However, longitudinal data measuring this process during adolescence is lacking.

Most of the literature in this field consists of small case–control or cohort studies^13,14^. The absence of cam morphology when the physis is open^13,14^, and high prevalence of cam morphology when hips are skeletally mature led to the proposal that cam morphology develops around the time of physeal closure^1,2,7,13,14^. Baseline data from our cohort demonstrated morphological changes consistent with early cam development in individuals 10 years of age^1,2,6,15^. Longitudinal data is required to understand the development of cam morphology within the individual^16^. Studies using radiography^2,6,7^ cannot assess the cartilaginous structures of skeletally immature hips. Control populations are also necessary to determine what threshold of activity predisposes to cam development.

The aim of this study was to perform 3-year longitudinal follow-up of a control population and elite athlete cohort to compare the change in hip and growth plate anatomy between athletes and controls.

## Methods

### Study design

Prospective cohort study.

### Ethical approval

Ethical approval was granted by Oxford University Medical Sciences Inter-Divisional Research Ethics Committee (MSDIDREC-C2-2013-11). All procedures performed in this study involving human participants were in accordance with the ethical standards of the institutional and/or national research committee and with the 1964 Helsinki Declaration and its later amendments or comparable ethical standards. Prior to participation in the study informed consent was obtained from all participants or parents or legal guardians if the participant was under 16 years of age.

### Population

Individuals aged 9–18 years were recruited from Southampton Football Club (SFC) Academy, Oxford United Football Club (OUFC) Academy, and local schools (controls) at baseline. Participant recruitment was as previously reported^15^. The control population was expected to include a wide range of activity levels. Male and female controls were recruited to try and provide a gender comparison for the control arm. However, a comparable age matched female cohort of academy football players was not available at the time of recruitment.

**Figure 1:**
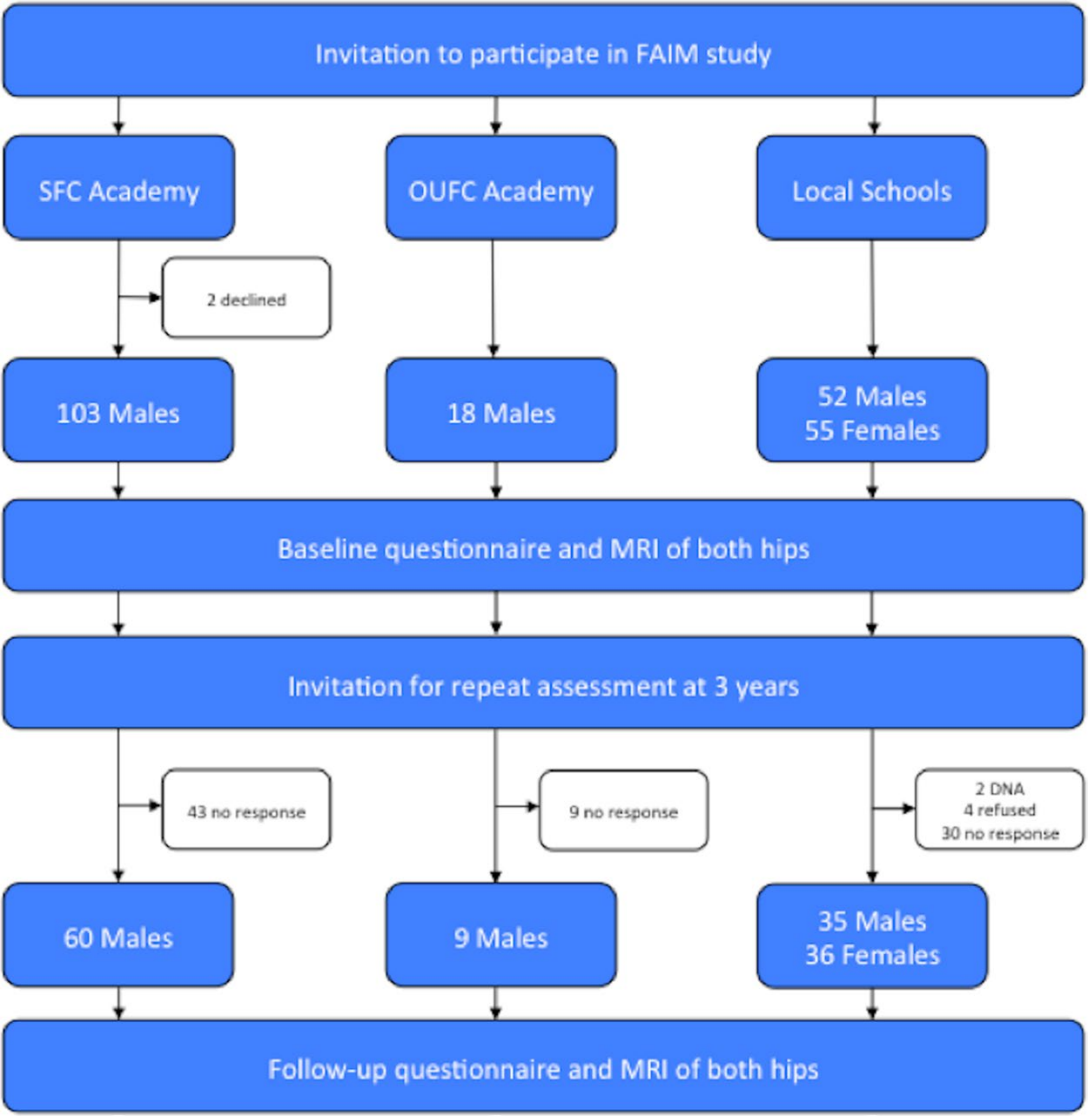
Recruitment flowchart for study.

Participants were invited for repeat assessment three years after initial baseline visit via post, email, and telephone (Fig. 1). Loss to follow-up was 42% for SFC, 50% for OUFC, and 34% for Controls. All football players who remained a member of the football club, but no player who had been released from the club attended follow-up. Four individuals from the control cohort declined repeat assessment, two did not attend their arranged follow-up appointment, and thirty individuals did not respond to invitation.

### Questionnaires

Activity was evaluated using two methods: (i) By cohort (elite footballer cohort versus general population controls); (ii) Using a patient reported outcome measure called the Physical Activity Questionnaire (PAQ). The PAQ is divided into use for older children (aged 9–13 years) and adolescents (aged 14–18 years)^17^. The PAQ collects information on sport and exercise undertaken during an average week. Multiple ordinal questions are summarized to a continuous mean score between 1 and 5. A score closer to 1 indicates low physical activity, whereas a score closer to 5 indicates high physical activity^18^.

Primary and secondary outcome measures were compared between cohorts to determine impact of playing elite level football during adolescence on change in hip and growth plate anatomy. The PAQ score was treated both as a continuous and ordinal variable. The PAQ score was used as a continuous variable to determine the relationship between activity level and changes in hip and growth plate anatomy. It was used as an ordinal variable to quantify impact of different levels of activity on outcome measures as follows; Individuals with a PAQ score between 1 and 2 (= PAQ 1–2), between 2 and 3 (= PAQ 2–3), between 3 and 4 (= PAQ 3–4), and between 4 and 5 (= PAQ 4–5).

### Imaging protocol

Cam morphology was assessed with MRI of both hips using a 3 T Philips Achieva platform and torso coil (Philips Healthcare). Two morphological sequences were acquired: three-dimensional (3D) water selective fluid (WATSf) to image joint cartilage and bone, and 3D proton density fat saturation (PDFS) to image the physeal scar (Supplementary file for sequence parameters. Note that Supplementary file has the same title and authors as the main manuscript). 3D multiplanar reconstructions were performed using OsiriX Software (V.6.0.2, Pixmeo). Radial images were acquired around the axis of the femoral neck at 30 degree intervals. The coronal axis (12 o’clock position) was positioned parallel to the axis of the proximal femur diaphysis^15^. MRI outcomes were incomplete due to claustrophobia or movement artifact in five hips at baseline (two participants in SFC cohort and one female control). The follow-up dataset was complete.

### Imaging measurements

Cam morphology was quantified using the alpha angle for cartilage (Supplementary Fig. 1). Cartilage alpha angle was selected because in skeletally immature hips the ossified regions of the femoral head do not reflect the overall hip shape (Supplementary Fig. 2). The primary outcome measure was change in mean cartilage alpha angle between baseline and follow-up. Epiphyseal morphology was quantified using epiphyseal extension (Supplementary Fig. 1)^19^, which has been shown to precede the development of cam morphology. Change in epiphyseal extension between baseline and follow-up was a secondary outcome measure. The ossification groove of Ranvier and the perichondrial fibrous ring of La Croix were quantified using a bespoke semi-automated methodology^20^ to segment the Fibrochondrooseous Tissue Area (FTA) at the femoral head-neck junction (Supplementary Fig. 3). Hypertrophy of this area has been suggested to precede cam formation^15^.

Alpha angle, epiphyseal extension, and FTA were measured using custom-developed software on radial slices at 11 o'clock, 12 o'clock, 1 o'clock, 2 o'clock and 3 o'clock. Alpha angle and epiphyseal extension were taken as an average from the values measured on each radial slice. The physis of each hip was scored as either open, partially closed, or closed (Supplementary Fig. 4). (See supplementary file for reproducibility results. Note that Supplementary file has the same title and authors as the main manuscript).

### Statistical analysis

Statistical calculations were performed using STATA V.14.1 (College Station, Texas, USA). Distribution of values was examined using histograms and kernel density plots. To account for selection bias introduced by loss to follow-up, inverse probability weighting with regression adjustment modelling^21^ was adopted to assess variables that predict change in alpha angle and change in epiphyseal extension. Multivariate linear regression modeling was used to evaluate the relationship between variables. Variables included in multivariate analysis were cohort, age, gender and activity level. Interactions were evaluated with linear regression of each combination of variables that predict average alpha angle and epiphyseal extension. None reached statistical significance; hence no interaction terms were included in the multivariate models. Statistical significance was set at p < 0.05.

### Power calculation

See supplementary data for power calculations.

## Results

### Participant demographics

At baseline there were 228 individuals (449 hip MRIs) (mean age 12.6 years, SD 2.6) and 140 individuals (280 hip MRIs) attended three-year follow-up (mean age 15.1 years, SD 2.7). Mean follow up was 3.0 years, SD 0.3 (Table 1).

**Table 1:** Cohort demographics.

		Baseline					Follow-up				
		Individuals (hips)	Open physis	Partially open physis	Closed physis	PAQ score, mean (± SD)	Individuals (hips)	Open physis	Partially open physis	Closed physis	PAQ score, mean (± SD)
**Male footballers**											
Age at baseline (years)	9–10	37 (73)	73	0	0	3.42 (± 0.43)	28 (56)	26	0	0	3.43 (± 0.86)
	11–12	32 (64)	64	0	0	3.36 (± 0.65)	22 (44)	58	0	0	3.39 (± 0.77)
	13–14	22 (44)	22	18	4	2.98 (± 0.73)	9 (18)	12	2	14	3.05 (± 0.32)
	15–16	20 (40)	2	10	28	2.51 (± 0.65)	7 (14)	0	0	12	2.93 (± 0.61)
	17–18	8 (16)	0	2	14	2.17 (± 0.40)	3 (6)	0	0	12	2.71 (± 0.23)
Total		119 (237)	161	30	46	3.11 (± 0.71)	69 (138)	96	2	40	3.29 (± 0.76)
**Male controls**											
Age at baseline (years)	9–10	15 (30)	30	0	0	3.10 (± 0.46)	12 (24)	12	0	0	3.19 (± 0.77)
	11–12	14 (28)	28	0	0	3.28 (± 0.55)	10 (20)	18	4	0	2.90 (± 0.81)
	13–14	8 (16)	12	2	2	2.87 (± 0.59)	4 (8)	8	2	6	2.12 (± 0.67)
	15–16	13 (26)	10	2	14	2.22 (± 0.64)	8 (16)	0	0	14	2.41 (± 0.87)
	17–18	2 (4)	0	0	4	2.14 (± 0.44)	1 (2)	0	0	6	3.17 (± 0)
Total		52 (104)	80	4	20	2.86 (± 0.69)	35 (70)	38	6	26	2.80 (± 0.86)
**Female controls**											
Age at baseline (years)	9–10	11 (22)	22	0	0	2.96 (± 0.51)	10 (20)	8	0	4	2.49 (± 0.80)
	11–12	6 (12)	10	2	0	2.72 (± 0.76)	5 (10)	2	2	10	2.00 (± 0.51)
	13–14	12 (24)	0	0	24	2.30 (± 0.56)	8 (16)	0	0	10	1.52 (± 0.38)
	15–16	19 (38)	0	0	38	2.14 (± 0.65)	9 (18)	0	0	18	1.77 (± 0.41)
	17–18	6 (12)	0	0	12	2.21 (± 0.58)	4 (8)	0	0	18	2.85 (± 0.96)
Total		54 (108)	32	2	74	2.42 (± 0.69)	36 (72)	10	2	60	2.07 (± 0.76)

### Risk factors for cam development

**Figure 2:**
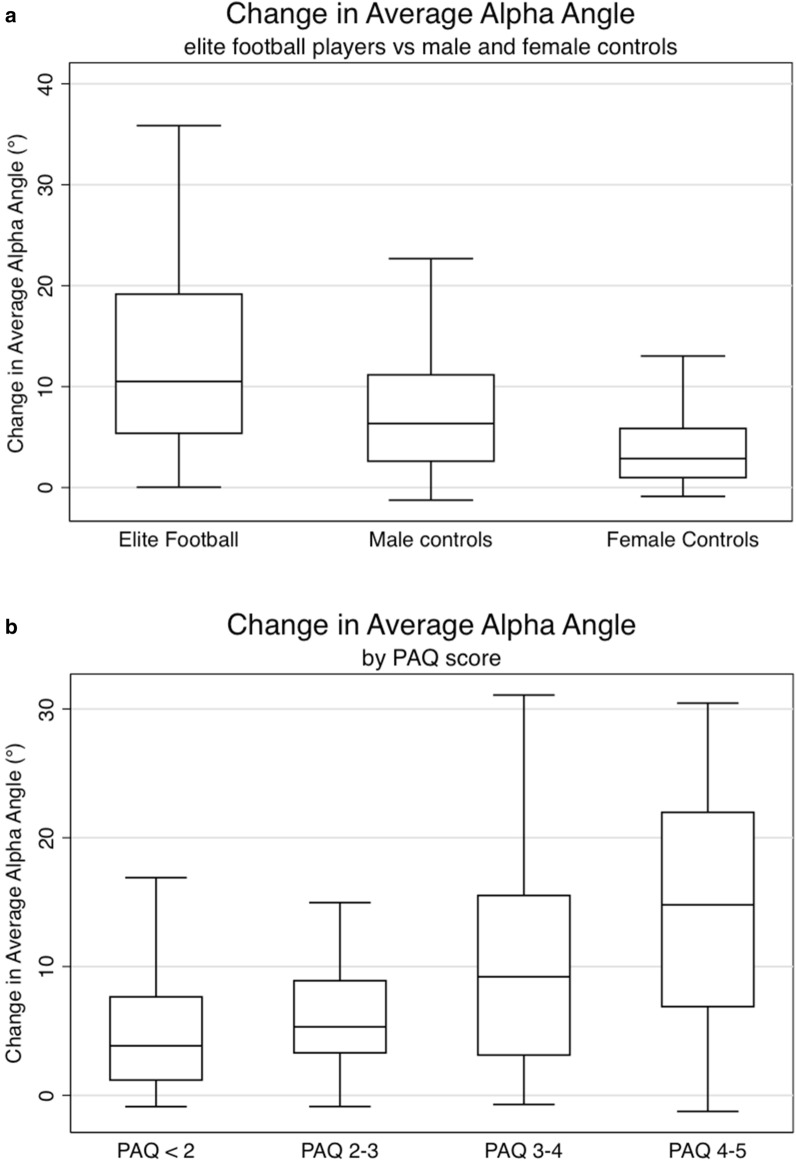
**(a)** Change in average cartilage alpha angle in individuals from the elite footballer players, male controls, and female controls. Box and whisker plot. **(b)** Change in average cartilage alpha angle by PAQ score at baseline. Box and whisker plot.

Elite football players had a significantly greater change in alpha angle than controls between baseline and follow-up (Fig. 2a). Adjusting for age, elite football players had a change in average alpha angle 4.83 degrees greater than male controls (p < 0.001). Male controls had a significantly greater change in alpha angle than female controls between baseline and follow-up. Adjusting for age male controls had a change in average alpha angle 2.27 degrees greater than female controls (p = 0.016) (Table 2).

**Table 2:** Predictors of change in average alpha angle.

		Hips	Mean change in average alpha angle (± SD)	Univariate regression			Multivariate regression 1*			Multivariate regression 2**			Multivariate regression 3***		
				Coefficient	95% CI	P value	Coefficient	95% CI	P value	Coefficient	95% CI	P value	Coefficient	95% CI	P value
Age at baseline	Months of age (continuous)	275	8.94 (± 7.79)	−0.11	−0.12 to −0.09	0.007	−0.01	−0.02 to −0.00	0.037	0.00	−0.00 to 0.01	0.285			
	9–10 years	97	11.37 (± 8.74)	8.00	5.95 to 10.08	< 0.001									
	11–12 years	72	12.63 (± 7.52)	9.27	7.21 to 11.33	< 0.001									
	13–14 years	42	5.39 (± 4.47)	2.03	0.29 to 3.78	0.022									
	15–16 years	48	3.45 (± 2.74)	0.10	−1.26 to 1.46	0.889									
	17–18 years	16	3.36 (± 3.37)	–	–	–									
Gender	Male	203	10.67 (± 8.05)	6.63	5.17 to 8.02	< 0.001				5.00	2.77 to 7.23	< 0.001	2.10	1.26 to 2.94	< 0.001
	Female	72	4.05 (± 4.13)	–	–	–				–	–	–			
Physis	Open	151	12.59 (± 8.26)	9.95	8.54 to 11.37	< 0.001							9.29	7.50 to 11.08	< 0.001
	Open-closed	54	6.90 (± 4.38)	5.69	3.94 to 7.44	< 0.001							5.36	3.32 to 7.40	< 0.001
	Closed	70	2.64 (± 2.32)	–	–	–							–	–	–
BMI		275	–	−0.77	−1.29 to −0.25	0.034									
Dominant leg	Dominant	152	9.28 (± 7.98)	0.77	1.07 to 2.60	0.413									
	Non dominant	123	8.52 (± 7.56)	–	–	–									
Cohort	Footballers	133	12.44 (± 8.45)	5.12	3.12 to 7.11	< 0.001	4.83	2.84 to 6.82	< 0.001						
	Male controls	70	7.32 (± 5.97)	–	–	–	–	–	–						
	Female controls	72	4.05 (± 4.13)	−3.28	4.96 to −1.59	< 0.001	−2.27	−4.12 to −0.43	0.016						
PAQ	Continuous			2.90	2.10 to 3.70	0.014				0.79	0.56 to 1.01	< 0.001	1.00	0.55 to 1.44	< 0.001
	< 2	68	5.74 (± 6.61)	–	–	–									
	2–3	72	7.06 (± 5.59)	1.32	−0.70 to 3.34	0.199									
	3–4	103	10.67 (± 8.22)	4.93	2.71 to 7.15	< 0.001									
	4–5	32	14.39 (± 8.76)	8.65	5.28 to 12.02	< 0.001									

*Age and cohort as covariables.

**Age, gender, and Physical Activity Questionnaire as covariables.

***Physeal maturity, gender, and Physical Activity Questionnaire as covariables.

Individuals with high activity levels (PAQ score) had a significantly greater change in alpha angle than those with lower activity levels (Fig. 2b). Adjusting for age and gender individuals with a PAQ score between 3 and 4 and between 4 and 5 had a change in average alpha angle 0.58 degrees (p = 0.008) and 3.20 degrees (p = 0.025) greater than individuals with a PAQ less than 2 respectively. Controlling for age and gender there was a positive linear relationship between PAQ score and change in average cartilage alpha angle (coefficient 0.79, p ≤ 0.001). (Table 2).

There was no significant association between leg dominance and change in average alpha angle. A significant association between BMI and alpha angle was observed in the univariate regression analysis, but this became insignificant when controlling for age, gender, and activity.

### Age of cam development

#### Age

The greatest change in alpha angle in all participants occurred in individuals aged 11–12 years at baseline. No significant difference was seen in change in alpha angle between age groups in individuals older than 14 years at baseline. The change in alpha angle observed in individuals older than 14 years was less than the smallest detectable difference (4.2 degrees, see supplementary data), suggesting no true morphological change occurred (Table 2). The greatest change in alpha angle in footballers and male controls occurred in individuals aged 11–12 years at baseline. The greatest change in alpha angle in female controls occurred in individuals aged 9–10 years at baseline (Table 3).

**Table 3:** Change in average alpha angle with age by cohort.

		Average alpha angle (± SD)			Univariate regression		
		Baseline	Follow-up	Mean change	Coefficient	95% CI	P value
**Male footballers**							
Age at baseline (years)	9–10	48.31 (± 4.04)	62.62 (± 10.47)	14.66 (± 9.61)	9.73	7.03 to 12.42	< 0.001
	11–12	50.37 (± 4.95)	65.31 (± 9.13)	15.03 (± 7.36)	10.09	7.74 to 12.45	< 0.001
	13–14	61.41 (± 8.801	66.06 (± 8.79)	8.17 (± 4.46)	3.24	1.07 to 5.41	0.003
	15–16	65.89 (± 12.55)	70.33 (± 12.55)	4.97 (± 2.34)	0.04	−1.42 to 1.49	0.963
	17–18	66.43 (± 15.57)	58.27 (± 7.49)	4.93 (± 1.16)	–	–	–
Average		55.28 (± 11.01)	64.52 (± 10.19)	12.44 (± 8.45)			
**Male controls**							
Age at baseline (years)	9–10	47.76 (± 3.00)	54.93 (± 5.28)	7.18 (± 5.88)	2.81	−0.40 to 6.02	0.047
	11–12	50.43 (± 4.82)	60.92 (± 8.34)	11.21 (± 6.84)	6.84	3.16 to 10.52	< 0.001
	13–14	56.75 (± 5.11)	62.10 (± 5.70)	6.08 (± 3.25)	1.70	−1.37 to 4.78	0.278
	15–16	60.29 (± 9.26)	65.81 (± 11.40)	3.67 (± 3.18)	−0.71	−3.41 to 2.00	0.609
	17–18	58.13 (± 2.80)	61.31 (± 6.34)	4.37 (± 2.29)	–	–	–
Average		53.40 (± 7.73)	60.13 (± 8.84)	7.32 (± 5.97)			
**Female controls**							
Age at baseline (years)	9–10	47.86 (± 3.83)	55.32 (± 4.97)	7.68 (± 5.00)	5.76	−1.59 to 1.92	< 0.001
	11–12	52.08 (± 5.42)	58.78 (± 5.59)	5.39 (± 3.42)	3.46	−1.84 to 1.83	0.007
	13–14	50.44 (± 4.76)	53.42 (± 4.80)	1.09 (± 2.16)	−0.00	0.94 to 5.99	1.000
	15–16	50.73 (± 7.01)	53.02 (± 7.39)	2.09 (± 1.95)	0.17	3.14 to 8.38	0.853
	17–18	54.34 (± 7.33)	58.18 (± 6.60)	1.93 (± 2.35)	–	–	–
Average		50.63 (± 6.06)	55.12 (± 6.13)	4.05 (± 4.13)			

#### Physeal maturity

Younger individuals with an open physis at both baseline and follow-up experienced the greatest change in alpha angle. Adjusting for activity level, age, and gender, individuals with an open physis at baseline and follow-up had a change in average alpha angle 9.29 degrees greater than individuals with a closed physis (p < 0.001). Individuals with a closed physis had a change in average alpha angle of 2.64 degrees between baseline and follow-up, which was not statistically significant and was less than the smallest detectable difference for intra- and inter-observer reproducibility measures for alpha angle. (Table 2).

### Location of cam development

The greatest change in average alpha angle occurred at the antero-superior (1 o’clock) femoral head-neck junction. Elite footballers had significantly greater change in average alpha angle than male controls at the 12, 1, 2, and 3 o’clock positions, but not at the 11 o’clock position, with the greatest difference at the 1 o’clock position. Adjusting for age elite footballers had a change in average alpha angle 8.18 degrees greater at the 1’clock position than male controls (p < 0.001) (Supplementary table 1).

### Pathogenesis of cam development

#### Lateral epiphyseal extension

A significant but weak positive correlation was seen between change in average alpha angle and change in average lateral epiphyseal extension (coefficient = 0.002, p = 0.012). This correlation was significant at the 12, 1, and 2 o’clock positions, but not at 11 and 3 o’clock. A significant positive correlation was also seen between average follow-up alpha angle and average follow-up lateral epiphyseal extension (coefficient = 0.002, p = 0.031). Adjusting for age elite footballers had a change in lateral epiphyseal extension 0.01 greater than male controls (p = 0.019). There was no statistical difference in change in lateral epiphyseal extension between male and female controls. (Supplementary table 2).

#### Fibrochondrooseous tissue area

A significant positive correlation was seen between baseline FTA and change in average alpha angle (coefficient 4.09, p = 0.003). This correlation was significant at the 11, 12, 1, 2, and 3 o’clock positions. Adjusting for age elite footballers had an FTA 28.92mm^2^ greater than male controls at baseline (p < 0.001). There was no statistical difference in FTA between male and female controls. (Supplementary table 3).

## Discussion

The aim of this study was to compare the change in hip and growth plate anatomy between athletes and controls. Identifying individuals at greatest risk of cam formation may allow the development of preventative strategies. This study demonstrates that males undertaking intense exercise between the ages of 11 and 14 years are at greatest risk of developing cam morphology.

Our results demonstrate that activity level and gender play a significant role in the development of cam morphology. The increase in alpha angle was 4.8 degrees greater in male footballers compared with male controls. This magnitude of morphological change during adolescence and greater difference in elite footballers is consistent with previously reported findings^1,14,22^. Tak et al.^22^ reported a significantly higher prevalence of cam deformity in elite footballers compared to amateur footballers. Carsen et al.^14^ found alpha angles to be 5 to 6 degrees higher in individuals with closed as opposed to open physes. Siebenrock et al.^1^ found a mean difference in alpha angle between athletes and controls of 6.2 degrees in individuals with an open physis and 16.9 degrees with a closed physis. Siebenrock et al.^1^ also reported the greatest difference in change in alpha angle to be at the 1 o’clock position, which was also seen in this study.

Higher PAQ scores were also associated with a greater change in alpha angle. Activity levels have previously been reported to be higher in individuals with cam morphology^14^. A dose response relationship between training frequency and cam morphology in football players has been described previously^15,22^. This study found individuals with a PAQ score above 3 to be at significantly elevated risk. Quantification of pathological levels and type of activity raises the possibility of introducing screening tools to identify individuals at risk of developing cam morphology. However, the PAQ score is limited in differentiating between active and inactive individuals^18^, but not type, intensity, or duration of activity. More precise quantification methods would be needed before an individual’s risk could be accurately stratified. As with previous studies, there was no difference in alpha angle measurements between the dominant and non-dominant leg.

Change in average alpha angle was significantly greater in male controls than female controls. Although males on average had a higher PAQ score, this was controlled for in the regression models. This finding is consistent with reports from other cohorts where there is a lower prevalence of cam morphology in female than male populations^4,23^. These findings suggest cam morphology may have a gender-specific pathogenesis, though further research comparing male and female elite athlete cohorts is required to fully elucidate this relationship.

Studies have demonstrated that cam morphology does not develop after skeletal maturity^16^. Similarly, in our study there was no statistically significant increase in alpha angle in individuals with a closed physis at baseline. Observed post physeal closure changes in alpha angle found in other studies are likely due to positional artefact in radiography, which is overcome by using MRI^7^.

In this study, the greatest change in alpha angle occurred between the ages of 9–10 years in females and 11–12 years in male controls and footballers. No significant change in average alpha angle occurred in male or female controls over 12 years or footballers over 14 years at baseline. This is consistent with our cross-sectional data from this cohort demonstrating no statistically significant increase in alpha angle beyond 14 years of age^15^. These findings support the proposal that cam morphology develops when hip development is responsive to load whilst the physis is open during active growth skeletal growth^15^.

Mechanical loading is recognised as a key factor for normal bone development^24^. Increased levels of sex steroids, growth hormone peaks, insulin like growth factors and genetic factors at the start of adolescence may result in increased plasticity under loading^25^. This could provide a possible explanation for cam morphology development at this early age in active individuals. Finite element models of the hip show high mechanical stress on impact loading precisely where a cam deformity most frequently develops^26^. Excessive loading at the femoral head-neck junction at this key development time may trigger abnormal or excessive bone development, resulting in cam morphology formation.

Cam development was associated with epiphyseal extension that was maximal at the 1 o’clock position. The relationship between epiphyseal extension and subsequent cam morphology is not well understood^7,12,15^. However, previous studies also report athletes show greater epiphyseal extension than controls^12^. Subtle morphological changes in physeal development have been reported in association with many sports^27^, including the proximal humeral physis in baseball^28^, and cam development is likely to represent a potentially modifiable physiological adaptation to load. A strong association was found between FTA and cam development. It may be that soft tissue hypertrophy and ossification at the femoral head neck junction represents a distinct and more likely mechanism for cam morphology formation than lateral epiphyseal extension.

The main limitation of this study is the loss to follow-up. This is a well recognized issue in elite sporting cohorts due to national and international relocation and low player retention^7^. However, our study remains adequately powered at the follow-up point with similar cohort demographics to baseline (Table 1).

A significant limitation of this study is the lack of an age matched elite female footballers cohort. At the time of recruitment an age and activity level matched cohort of elite female footballers did not exist within the football academy structure. As such, we are unable to fully elucidate the effect of gender on cam morphology formation in this study. The decision to include the female control cohort data was based on two main reasons; (i) The female control group provides an invaluable dataset that may allow comparison to any future work performed with adolescent elite female athletes. (ii) The lack of availability of an elite female footballer cohort highlights the disparity between genders in elite sports academies and sports science research, and demonstrates this as an area that must be addressed in future research. Although we have included female control data, this study avoids any direct comparisons between elite male footballers and female controls.

A further limitation is that the control group may not be representative of the general population due to participation bias in sports and exercise projects. Additionally, activity levels may have varied on an individual basis between baseline and follow up. However, no significant difference was seen in PAQ scores between time points. Further limitations apply to using the alpha angle as a morphological measure. There exists no universally agreed diagnostic threshold for cam morphology and a quantitative value for pathological change in alpha angle is unknown^19^. Moreover, hips may undergo further epiphyseal extension resulting in worsening asphericity without a rise in alpha angle. Follow up of this cohort beyond full skeletal maturity may determine what constitutes a pathological change in femoral morphology.

In conclusion, the development of cam morphology is greatest age 9–10 in females and 11–12 in males. There is unlikely to be significant change in alpha angle beyond 14 years of age. Physical activity during adolescence is strongly associated with cam development in males, and quantification of pathological physical activity levels and type warrants further exploration as a potential screening tool for individuals at greatest risk of developing cam morphology. Hypertrophy of the Fibrochondroosseous Tissue Area followed by epiphyseal extension is likely to represent physiological adaptation to load and be the mechanism by which cam morphology forms. Potential interventions to prevent or modify cam morphology formation could include training load modification in elite adolescent football players younger than 14 years. There may also be an argument for avoiding early specialization in sports that subject the developing physis to repetitive loading due to frequent changes in velocity. Moreover, active monitoring of hip anatomy for changes at the lateral femoral head-neck junction may be of some benefit in identifying at risk individuals.

## Supplementary Information

Supplementary Information.

## Data Availability

Data is available from the corresponding author upon reasonable request.
