# Supplementary material for: A longitudinal cohort study of adolescent elite footballers and controls investigating the development of cam morphology
Source: Sci Rep. 2021 Sep 17;11:18567. doi: 10.1038/s41598-021-97957-2 (PMC8448877; doi:10.1038/s41598-021-97957-2)
Supplement: Supplementary file 1 — Supplementary Information. [file 41598_2021_97957_MOESM1_ESM.docx]

**A Longitudinal Cohort Study of Adolescent Elite Footballers and Controls Investigating The Development of Cam Morphology**

**AUTHOR LIST**

1. Scott Fernquest^1^ (scott.fernquest@gmail.com), Botnar Research Centre, Old Road, Oxford, OX3 7LD. Tel: 01865 227374*
2. Antony Palmer^1^ (antony.palmer@ndorms.ox.ac.uk)
3. Mo Gimpel^2^ (mgimpel@saintsfc.co.uk)
4. Richard Birchall^2^ (rbirchall@saintsfc.co.uk)
5. John Broomfield^1^ (john.broomfield@ndorms.ox.ac.uk)
6. Thamindu Wedatilake^2^ (thamindu@hotmail.com)
7. Hendrik Dijkstra^3^ (hendrik.dijkstra@conted.ox.ac.uk)
8. Joanna Burchall^1^ (joanna.burchall@ouh.nhs.uk)
9. Thomas Lloyd^1^ (tomlloyd91@gmail.com)
10. Claudio Pereira^1^ (claudio.pereira@ndorms.ox.ac.uk)
11. Simon Newman^1^ (mrsimonnewman@gmail.com)
12. Andrew Carr^1^ (andrew.carr@ndorms.ox.ac.uk)
13. Sion Glyn-Jones^1^ ([sion.glyn-jones@ndorms.ox.ac.uk](mailto:sion.glyn-jones@ndorms.ox.ac.uk))

^1^Nuffield Department of Orthopaedics, Rheumatology, and Musculoskeletal Sciences, University of Oxford, Oxford, UK

^2^Southampton Football club, Southampton, UK

^3^Aspetar, Qatar Orthopaedic and Sports Medicine Hospital, Doha, Qatar; Weill Cornell Medicine Qatar; Department of Continuing Education, University of Oxford, Oxford, UK.

**SUPPLEMENTARY DATA**

**Power calculation**

The primary outcome measure was change in average alpha angle at the anterosuperior femoral head-neck junction measured on MRI radial slices. Sample size calculations were based on existing literature at 80% power and 5% significance using a two-sided t-test, where only one hip was considered for each participant. The desired detectable difference and standard deviation are based on comparable cohorts. In order to investigate the age of cam morphology development, the study sought to detect differences in alpha angle pre and post physeal closure in an athlete cohort and a general population control cohort. Carsen et al. reported a mean change in alpha angle of 4.85 degrees (SD 7.05) between individuals with an open and closed physis[1]. We calculated that a minimum of 35 individuals was required either side of physeal closure to study morphological changes during adolescence. This study also aimed to investigate differences in the alpha angle between the athlete cohort and general population controls. Siebenrock et al. reported a mean difference in alpha angle between athletes and controls of 6.2 degrees (SD 7.4) in individuals with an open physis and 16.9 degrees (SD 7.6) with a closed physis.[2] We calculated that a minimum of 24 individuals pre-physeal closure and 5 individuals post-physeal closure were required in the male control group for comparisons with male athletes. Cohorts were loaded for younger age groups to: i) Ensure an adequate number of individuals have an open physis to satisfy the power calculations. ii) Enhance the longitudinal component of the study as drop-out rates can be as high as 30% in athletic cohorts due to national and international relocation to continue their careers[3]. This is more common in players over the age of 16 on completion of their education. A 1:1 recruitment strategy was adopted for male and female controls.

**MRI Protocols:**

1. 3D Water Selective Fluid (WATSf): Sequence variant = 3D Gradient/Fast Field Echo with binomial pulse (1:3:3:1); Repetition time (TR) = 13.65ms; Echo time (TE) = 6.9ms; excitation Flip angle = 30 degrees; bandwidth = 145Hz/pixel; interpolated voxel size 0.29mm x 0.29mm x 0.4mm; averages= 2; acquired in true sagittal orientation.
2. 3D Proton Density with Fat Saturation (PDFS): Sequence variant = 3D VISTA, Spin Echo; Repetition time (TR) = 1300ms; Echo time (TE) = 40ms; excitation Flip angle = 180 degrees; refocusing Flip angle =35 degrees; bandwidth 243Hz/pixel; interpolated voxel size 0.625mm x 0.625mm x 0.65mm; averages= 2; acquired in true sagittal orientation

**Reproducibility**

All reproducibility measures were taken from the MRI radial planes of 20 hips (10 with open physis and 10 with closed physis). The Intraclass coefficient (ICC), Smallest Detectable Difference (SDD, mean change ± 1.96 x SD of the change), and Root Mean Square Coefficient of Variance (RMSCoV, root mean square of the change divided by the mean, multiplied by 100)[4] was measured for all variables.

*Alpha angle*

Intra observer ICC was 0.996 (95% CI:0.995 – 0.997), SDD was 3.388 degrees, and RMSCoV was 2.999%. Inter-observer ICC 0.914 (95% CI:0.866 – 0.945), SDD was 4.219 degrees, and RMSCoV was 4.606%.

*Lateral Epiphyseal Extension*

Intra observer ICC 0.998 (95% CI:0.997 – 0.0.999), SDD was 0.012, and RMSCoV was 0.864%. Inter-observer ICC 0.988 (95% CI: 0.982 – 0.993), SDD was 0.014, and RMSCoV was 1.092%.

*Fibrochondroosseous Tissue Area*

Intra observer ICC was 0.957 (95% CI: 0.940 – 0.974), SDD was 10.027, and RMSCoV was 0.597%. Inter-observer ICC was 0.934 (95% CI: 0.908 – 0.960), SDD was 12.323, and RMSCoV was 0.739%.

Supplementary Figure 1. Elite level football player age 11 years at baseline with soft tissue hypertrophy at the femoral head-neck junction (A) which preceded lateral epiphyseal extension and osseous cam morphology seen at follow-up when aged 14 years (B). Alpha angle was calculated by drawing a line from the centre of a best-fit circle surrounding the femoral head to the midpoint of a line transecting the narrowest portion of the femoral neck. A further line was then drawn from the centre of the best-fit circle to where the contour of the femoral head first exits this circle. The alpha angle is the angle between these two lines. Alpha angle shown in an elite level football player at baseline (C) and at follow-up (D). Epiphyseal extension was quantified by measuring the distance from the medial femoral head to the most distal extent of the epiphysis along a line parallel to the axis of the femoral neck as was created when measuring the alpha angle. This distance was then divided by the diameter of the femoral head to produce a standardised ratio. Lateral epiphyseal extension shown in an elite level football player at baseline (E) and at follow-up (F).


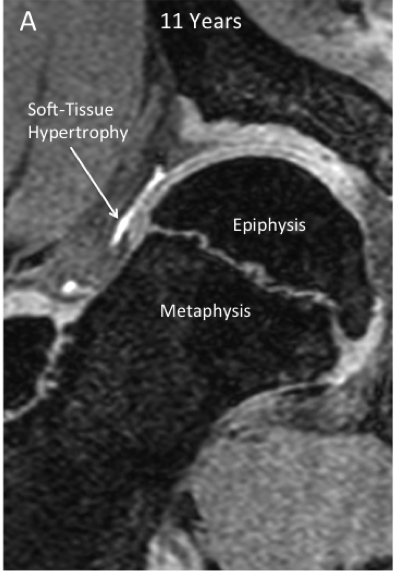

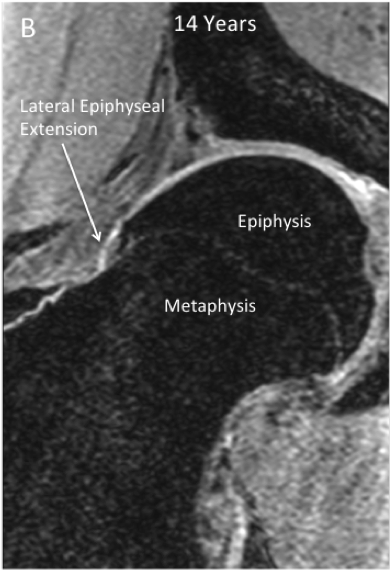


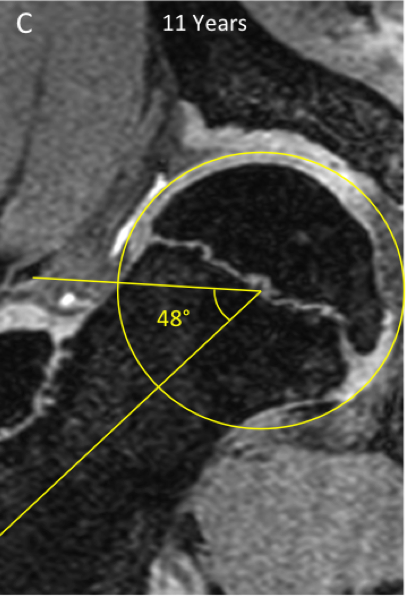

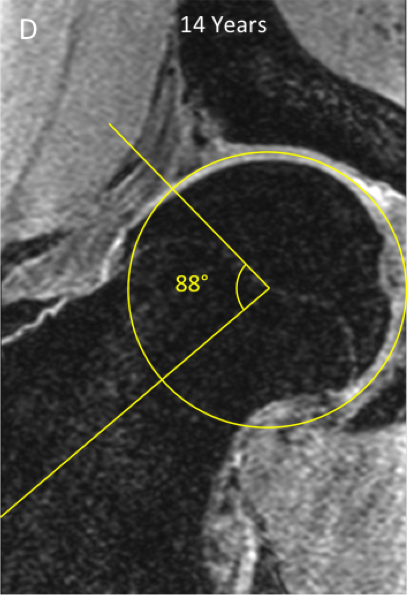


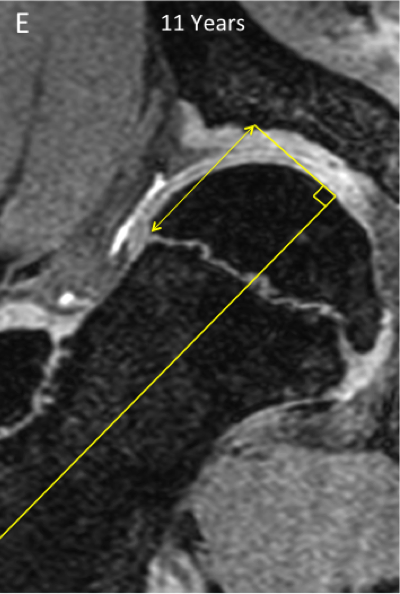

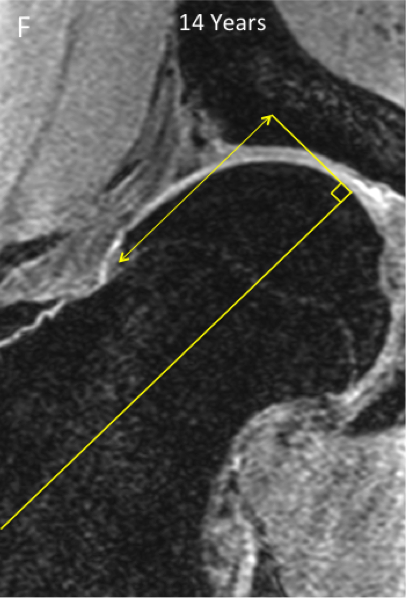


Supplementary Figure 2. Bone (A) and cartilage (B) alpha angle measurements in a ten year old male. Note the artificially raised alpha angle in the bone measurement due to the relatively small epiphysis compared to the metaphysis.


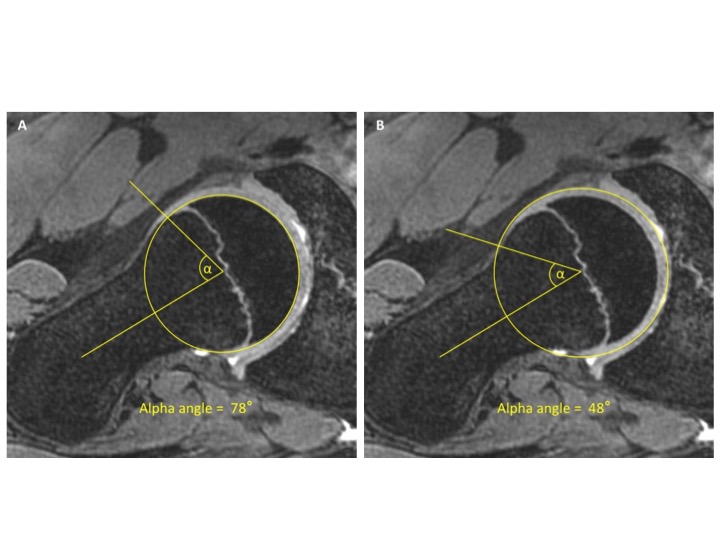


Supplementary Figure 3. Area of Fibrochondroosseous Tissue shown in an elite level football player at baseline age 12 years (A), with hierarchical partitioned terrain map overlaying the radial image and user selection of the Fibrochondroosseous Tissue Area (B).


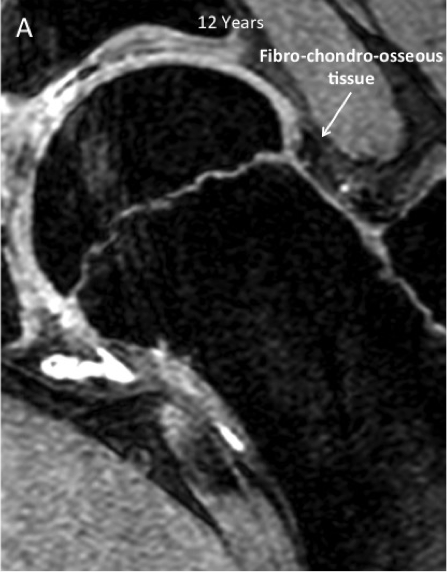

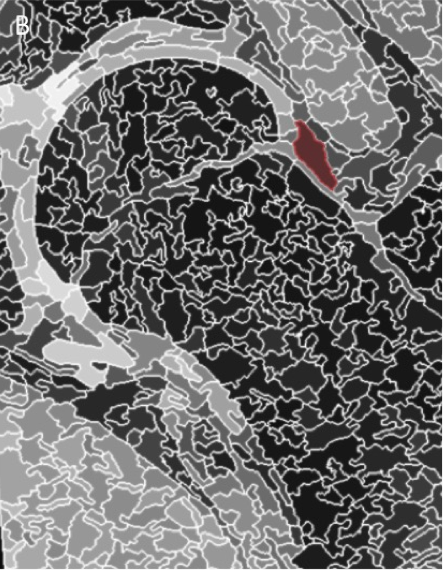


Supplementary Figure 4. Appearances of an Open, Partially Closed, and Closed Physis on WATSf MRI Sequence. A physis was deemed partially closed when there was contact between the epiphysis and metaphysis but the physis remained visible on the WATSf MRI sequence. When considering physeal closure as a binary variable, a partially closed physis was considered closed. A: Open Physis B: Partially Closed Physis C: Closed Physis.


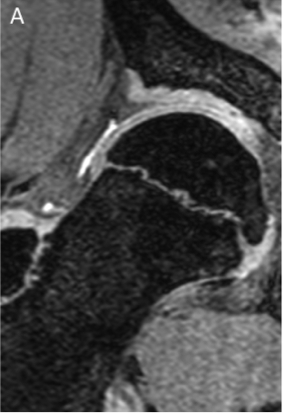

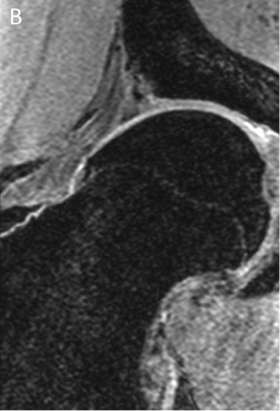


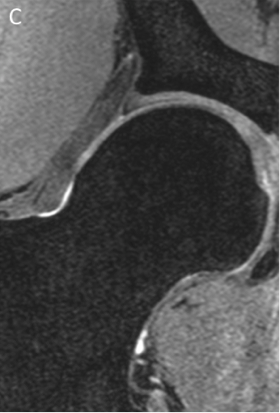


| **Position on femoral head-neck axis** | **Cohort** | **Change in average alpha angle (±SD)** | **Univariate Regression** | | | **Multivariate Regression*** | | |
| --- | --- | --- | --- | --- | --- | --- | --- | --- |
|  |  |  | **Coefficient** | **95% CI** | **p Value** | **Coefficient** | **95% CI** | **p Value** |
| **11 o’clock** | **Male**  **footballers** | 13.459 (±15.311) | -0.098 | -4.183 to 3.987 | 0.962 | -0.666 | -4.822 to 3.490 | 0.754 |
|  | **Male**  **controls** | 13.557 (±13.575) | - | - | - | - | - | - |
|  | **Female**  **controls** | 3.694 (±7.941) | -9.863 | -13.508 to -6.218 | <0.001 | -9.665 | -13.764 to -5.565 | <0.001 |
| **12 o’clock** | **Male**  **footballers** | 15.546 (±16.593) | 6.610 | 2.673 to 10.546 | 0.001 | 6.194 | 2.122 to 10.267 | 0.003 |
|  | **Male**  **controls** | 8.937 (±11.855) | - | - | - | - | - | - |
|  | **Female**  **controls** | 6.685 (±10.064) | -2.252 | -5.848 to 1.344 | 0.220 | -0.591 | -4.878 to 3.696 | 0.787 |
| **1 o’clock** | **Male**  **footballers** | 14.961 (±15.572) | 8.577 | 5.465 to 11.689 | <0.001 | 8.184 | 5.068 to 11.300 | <0.001 |
|  | **Male**  **controls** | 6.384 (±7.108) | - | - | - | - | - | - |
|  | **Female**  **controls** | 3.433 (±6.715) | -2.951 | -5.210 to -0.691 | 0.010 | -1.865 | -4.858 to 1.128 | 0.222 |
| **2 o’clock** | **Male footballers** | 12.820 (±11.879) | 7.405 | 4.795 to 10.015 | <0.001 | 6.936 | 4.423 to 9.450 | <0.001 |
|  | **Male**  **controls** | 5.415 (±7.153) | - | - | - | - | - | - |
|  | **Female**  **controls** | 2.907 (±5.471) | -2.515 | -4.591 to -0.424 | 0.018 | -1.225 | -3.618 to 1.169 | 0.316 |
| **3 o’clock** | **Male**  **footballers** | 8.542 (±8.329) | 4.053 | 2.112 to 5.994 | <0.001 | 3.697 | 1.761 to 5.634 | <0.001 |
|  | **Male**  **controls** | 4.489 (±5.734) | - | - | - | - | - | - |
|  | **Female**  **controls** | 2.825 (±4.189) | -1.663 | -3.307 to -0.020 | 0.047 | -0.781 | -2.496 to 0.934 | 0.372 |

Supplementary table 1. Change in alpha angle by position on femoral head-neck axis

*Age as a covariable

Supplementary table 2. Multivariate regression analysis of lateral epiphyseal extension with alpha angle by position on femoral head-neck axis. Age, gender, and activity level as covariables

| **Position on femoral head-neck axis** | **Regression with change in alpha angle*** | | | **Regression with follow up alpha angle*** | | |
| --- | --- | --- | --- | --- | --- | --- |
|  | **Coefficient** | **95% CI** | **p Value** | **Coefficient** | **95% CI** | **p Value** |
| **Average** | 0.002 | 0.001 to 0.002 | 0.012 | 0.002 | 0.001 to 0.003 | 0.031 |
| **11 o’clock** | 0.000 | -0.000 to 0.002 | 0.116 | 0.001 | -0.001 to 0.002 | 0.084 |
| **12 o’clock** | 0.001 | 0.000 to 0.001 | 0.020 | 0.001 | 0.001 to 0.002 | 0.016 |
| **1 o’clock** | 0.002 | 0.001 to 0.003 | 0.034 | 0.002 | 0.002 to 0.002 | 0.004 |
| **2 o’clock** | 0.001 | 0.001 to 0.002 | 0.009 | 0.002 | 0.001 to 0.002 | 0.006 |
| **3 o’clock** | 0.000 | -0.001 to 0.001 | 0.254 | 0.002 | 0.001 to 0.003 | 0.019 |

Supplementary table 3 - Multivariate regression analysis of Fibrochondroosseous Tissue Area with change in alpha angle by position on femoral head-neck axis. Age, gender, and activity level as covariables

| **Position on Femoral Head-Neck Axis** | **Regression of FTA with Change in Alpha Angle** | | |
| --- | --- | --- | --- |
|  | **Coefficient** | **95% CI** | **p Value** |
| **Average** | 4.09 | 3.86 to 4.33 | 0.003 |
| **11 o’clock** | 2.96 | 2.38 to 3.54 | 0.010 |
| **12 o’clock** | 3.21 | 1.45 to 4.98 | 0.027 |
| **1 o’clock** | 3.24 | 1.53 to 4.96 | 0.027 |
| **2 o’clock** | 2.85 | 0.24 to 5.47 | 0.046 |
| **3 o’clock** | 4.16 | 1.09 to 47.23 | 0.037 |

**REFERENCES**

1. Carsen S, Moroz PJ, Rakhra K, Ward LM, Dunlap H, Hay JA, et al. The Otto Aufranc Award. On the etiology of the cam deformity: a cross-sectional pediatric MRI study. Clin Orthop Relat Res. 2014;472(2):430-6.

2. Siebenrock KA, Ferner F, Noble PC, Santore RF, Werlen S, Mamisch TC. The cam-type deformity of the proximal femur arises in childhood in response to vigorous sporting activity. Clin Orthop Relat Res. 2011;469(11):3229-40.

3. Agricola R, Heijboer MP, Ginai AZ, Roels P, Zadpoor AA, Verhaar JA, et al. A cam deformity is gradually acquired during skeletal maturation in adolescent and young male soccer players: a prospective study with minimum 2-year follow-up. Am J Sports Med. 2014;42(4):798-806.

4. Surowiec RK, Lucas EP, Ho CP. Quantitative MRI in the evaluation of articular cartilage health: reproducibility and variability with a focus on T2 mapping. Knee surgery, sports traumatology, arthroscopy : official journal of the ESSKA. 2014;22(6):1385-95.
